# Perspectives on the prediction of catastrophic slope failures from satellite InSAR

**DOI:** 10.1038/s41598-019-50792-y

**Published:** 2019-10-01

**Authors:** Tommaso Carlà, Emanuele Intrieri, Federico Raspini, Federica Bardi, Paolo Farina, Alessandro Ferretti, Davide Colombo, Fabrizio Novali, Nicola Casagli

**Affiliations:** 1 Department of Earth Sciences, University of Florence, Via Giorgio La Pira 4, 50121 Florence, Italy; 2 Geoapp s.r.l., Via Francesco Veracini 30/G, 50144 Florence, Italy; 3 TRE ALTAMIRA, Ripa di Porta Ticinese 79, 20143 Milan, Italy

**Keywords:** Natural hazards, Hydrogeology

## Abstract

We demonstrate the potential of satellite Interferometric Synthetic Aperture Radar (InSAR) to identify precursors to catastrophic slope failures. To date, early-warning has mostly relied on the availability of detailed, high-frequency data from sensors installed *in situ*. The same purpose could not be chased through spaceborne monitoring applications, as these could not yield information acquired in sufficiently systematic fashion. Here we present three sets of Sentinel-1 constellation images processed by means of multi-interferometric analysis. We detect clear trends of accelerating displacement prior to the catastrophic failure of three large slopes of very different nature: an open-pit mine slope, a natural rock slope in alpine terrain, and a tailings dam embankment. We determine that these events could have been located several days or weeks in advance. The results highlight that satellite InSAR may now be used to support decision making and enhance predictive ability for this type of hazard.

## Introduction

Landslides occur in a wide variety of forms and environments. These are a direct expression of the geology, rheology, and destabilizing forces of the slope. The destructive power of a landslide, among other factors, is strictly related to the variation of available frictional strength, which in turn dictates how the rate of displacement changes with time. In particular, landslides prone to abrupt drops in shear resistance over one or more surfaces of rupture pose a major threat to vulnerable communities. Precursory signs may not be obvious and evacuation times are virtually inexistent once the failure paroxysmal phase is initiated. Therefore, prediction and early warning are the only viable options^1^. In this sense, Voight’s materials failure relation of tertiary creep under constant applied stress and temperature has found wide acknowledgement in the field of slope failure prediction^2,3^. Such empirical relation is linked to the theory of damage accumulation, and in particular to mechanisms of creep fracture by stress corrosion and power law lattice deformation^4^. Sub-critical crack nucleation and growth, which may be catalyzed by pore water pressure buildup, ultimately leads to a degree of voids coalescence that can no longer be supported by the remaining cross-sectional intact patches along the joint surface^5,6^. This induces a sudden transition from peak to residual strength conditions and the kinematic release of the unstable mass. The abovementioned processes are explicated by a phase of progressive deformation (i.e. accelerating or tertiary creep), during which strain increments of the slope surface up to failure are observed in the form a power-law^2,3^.

1 $${\ddot{\Omega }}^{-\alpha }\ddot{\Omega }-A=0$$

from which a linear law relating inverse velocity and time can be derived

2 $${\ddot{\Omega }}^{-1}={[A(1-\alpha )(t-{t}_{0})+{\dot{\Omega }}_{0}^{1-\alpha }]}^{1/(\alpha -1)}$$

where Ω is the measurable quantity (e.g. displacement), whilst *α* and *A* are empirical constants. Several authors have suggested that linearly extrapolating the theoretical time of singularity in an inverse velocity versus time plot (i.e. *α* = 2) can be used to predict the time of slope failure^7–10^. For this reason, monitoring activities are mostly focused on measuring the movement of the ground surface. Ground-based techniques used for failure prediction purposes include extensometers, distometers, survey stations and prisms, and slope stability radar. Nonetheless, many slope failures still come as a surprise because of the inability to effectively detect precursory tertiary creep. This often stems from: inadequate field of view of the instrument; limited number of measuring points; lack of ancillary data supporting the installation of a monitoring network; unawareness about the presence of ongoing instability phenomena; difficult site accessibility; economical or logistical constraints in general.

Such limitations may be largely solved through the exploitation of spaceborne platforms. In particular, satellite Interferometric Synthetic Aperture Radar (InSAR) has proved to be a unique tool for surface deformation monitoring. By calculating the deformation-induced phase shift of the back-scattered microwave signal between two coherent acquisitions, millimetric measurement accuracy and metric spatial resolution are attained in most atmospheric conditions, with no need to install physical reflectors on the ground. Earthquakes, volcanic activity, glacier motion, and subsidence have been among the most investigated topics^11^. However, the poor revisit capacity of orbiting satellites, the limited data accessibility, and the policy on image acquisition (i.e. background and on demand acquisitions) have so far prevented the use of satellite InSAR as a tool for systematic monitoring of critically unstable slopes. Recent developments have now opened up to the prospect of extending the application of satellite InSAR also to the field of slope failure prediction^12^. Most of the new missions consist in fact of an integration of more than one satellite working in constellation mode. This has significantly improved the frequency and regularity of the acquisitions, as well as the ground visibility of the flyovers. In addition, the newest algorithms for the processing of interferometric data-stacks make it possible to retrieve a greater amount of radar targets within the sensor swath. The interferometric products acquired by the Sentinel-1 constellation ensure a worldwide coverage and are freely distributed to the public.

## Results

We highlight the results of the processing of three stacks of Sentinel-1 images by means of the SqueeSAR algorithm^13^, with the aim of identifying precursory accelerating displacements over as many recent catastrophic slope failures. All these events were unforeseen, and caused multiple fatalities and/or massive economic losses. While data are here reviewed in retrospect, Raspini *et al*.^14^ described how to set up a systematic processing chain of Sentinel-1 interferometric data stacks, hence demonstrating the possibility of moving from a static analysis of archive images to a dynamic, continuously updated monitoring of the ground deformation. Such an approach could have been applied in the presented scenarios. The value of the results is enhanced by the fact that slope types of very different nature are considered, specifically: the failure of an open-pit mine slope, of a natural rock slope in alpine terrain, and of a tailings dam embankment. After a brief description of the case studies and of the monitoring data, we discuss the predictability of the failures and the key features of the precursors. It should be noted that the timing of the events and of the satellite acquisitions are expressed in terms of the respective local time zone.

### Open-pit failure

At about 8:41 pm on 17 November 2016, a catastrophic slope failure occurred in a copper open-pit mine (name and location may not be disclosed for confidentiality reasons). The incident caused the death of 16 mine workers and the termination of the extraction activities. As previously described by Carlà *et al*.^15^, the failure happened without apparent warning signs, as it mostly affected a sector of natural slope above the mine crest and outside of the field of view of the slope monitoring system in place at the site. The approximately 640 000 m^3^ unstable mass rapidly slid downslope and buried the uppermost benches of the pit, where production works were being carried out. The basal rupture surface was identified in proximity of the interface between a layer of recrystallized limestone blocks and underlying spilite rock formations included in the ore body, at an average depth of 11 m. The instability may be associated with a simple translational mechanism, and was reportedly driven by a period of unusual adverse weather conditions for the local climate (170 mm of rainfall between 25 October and 2 November 2016)^15^.

**Figure 1:**
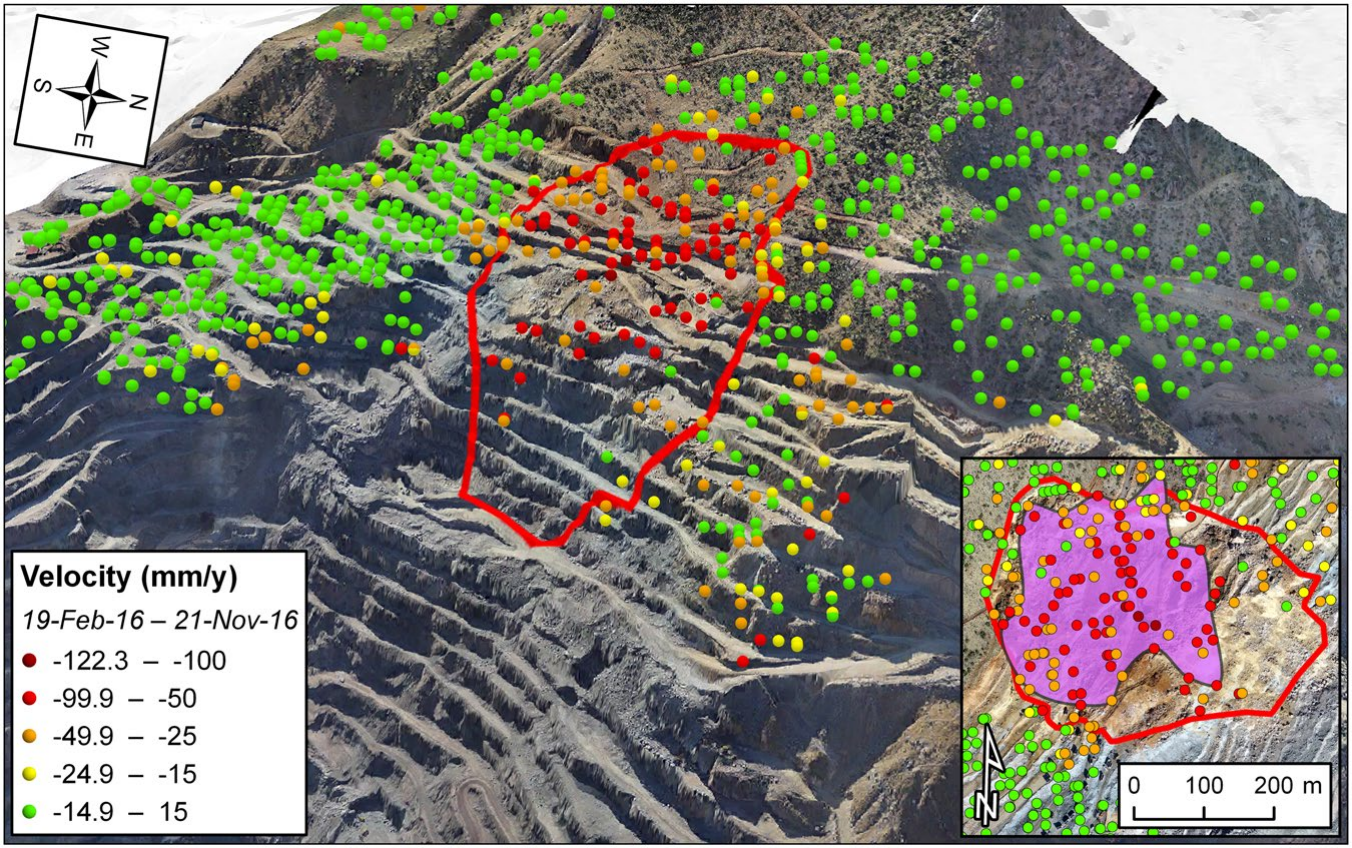
Satellite InSAR data showing precursory deformation leading up to the failure of the investigated open-pit mine slope on 17 November 2016. The purple-colored polygon in the inset delimits the area affected by accelerating trends of displacement. The underlying photos in the main figure and in the inset depict pre- and post-failure instants, respectively. The maps with satellite imagery were created with the ArcGIS PRO 2.4 software (https://www.esri.com/en-us/arcgis/products/arcgis-pro/overview).

28 SAR images have been acquired in ascending orbit over the mine site between 19 February and 21 November 2016 (i.e. four days after the event). The revisit time, which was of 12 days during the first part of the monitoring interval, diminished to 6 days in September 2016 as the second satellite of the Sentinel-1 constellation became fully operational. The last image prior to the failure was thus acquired on 15 November 2016. The distribution of the radar targets exhibiting precursory ground deformation matches remarkably the source area of the failure, with average line-of-sight (LOS) velocities for the monitoring interval generally ranging from 50 mm/y to 122.3 mm/y (Fig. 1). Up to 30.2 mm of LOS displacement were recorded in the period 9–15 November 2016. By contrast, radar targets surrounding the failure area were largely stable.

### Xinmo landslide (Sichuan Province, China)

A catastrophic 13 × 10^6^ m^3^ rock avalanche devastated the village of Xinmo at 5:45 am on 24 June 2017. The sliding mass, coming to rest over an area of about 1.5 km^2^, buried 62 houses and killed more than 100 people. The site falls within a well-known tectonically active region; even if repeated seismic events may have been responsible for a progressive destabilization of the rock mass, earthquakes are not considered the primary trigger of the failure^16^. The rupture surface developed in a heavily jointed bedrock mainly composed of metamorphic sandstone and phyllite. It has been proposed that the magnitude of the event was the consequence of a complex triggering mechanism, involving the sequential mobilization of different unstable slope sectors and the entrainment of older landslide deposits^17^. Intrieri *et al*.^18^ documented that the remoteness of the failure source area, located on a steep (55°–60°) alpine slope at an elevation of 3400 m a.s.l., meant that detecting precursory signs by means of conventional techniques was virtually impossible. For the same reason, the presence of tension cracks and the occurrence of minor precursory rockfalls were entirely unnoticed.

**Figure 2:**
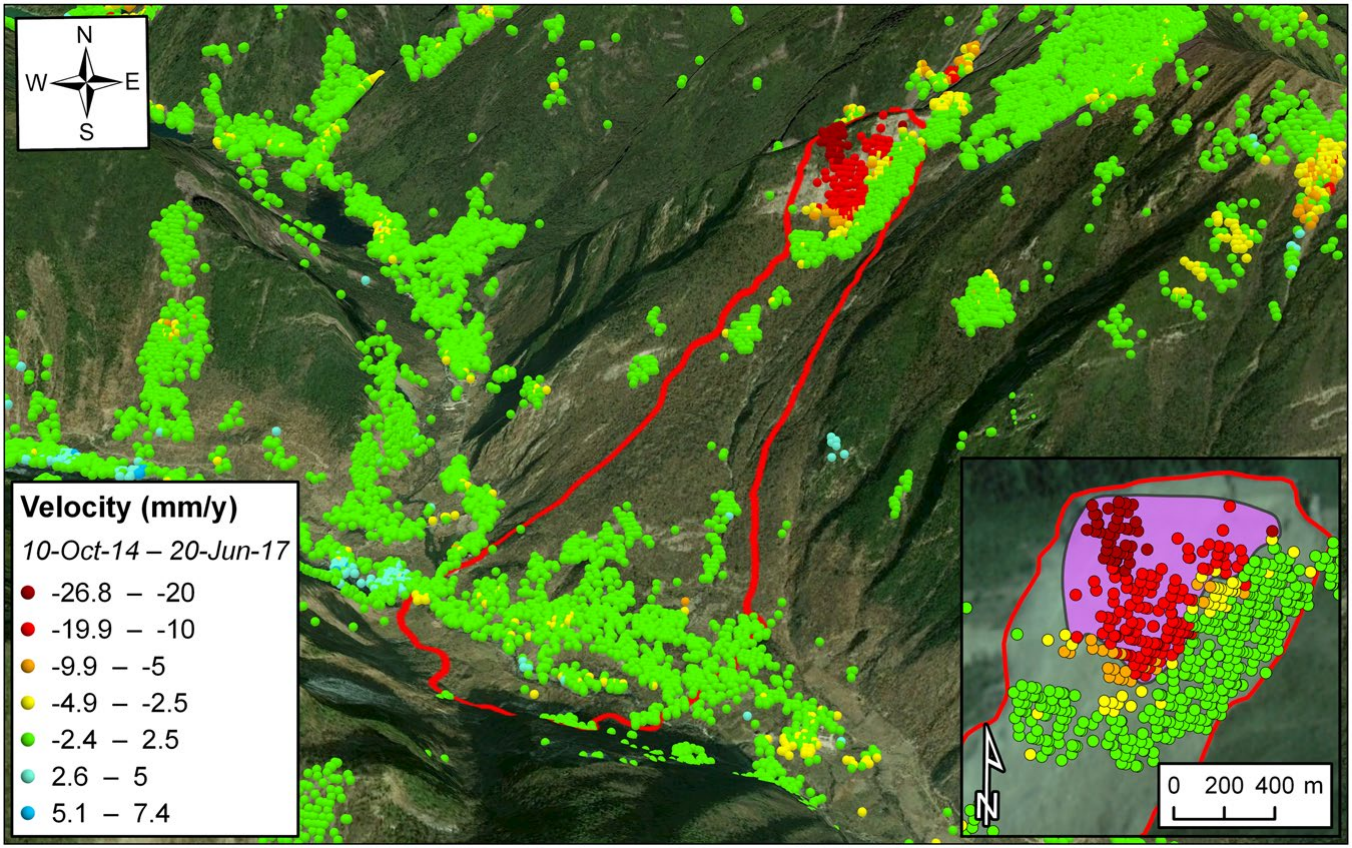
Satellite InSAR data showing precursory deformation leading up to the Xinmo landslide on 24 June 2017. The purple-colored polygon in the inset delimits the area affected by accelerating trends of displacement. The underlying photos in the main figure and in the inset depict pre- and post-failure instants, respectively. The maps with satellite imagery were created with the ArcGIS PRO 2.4 software (https://www.esri.com/en-us/arcgis/products/arcgis-pro/overview).

45 SAR images have been acquired in descending orbit between 10 October 2014 and 20 June 2017 (i.e. 4 days before the event), with a revisit time of 12 days. More than 700 radar targets have been identified within the boundaries of the landslide. Those located in the uppermost hillslope portion near the mountain crest recorded the largest average LOS velocities for the monitoring interval (generally ranging from 10 mm/y to 26.8 mm/y), and well outline the failure source area (Fig. 2). LOS displacements of up to 30.7 mm were recorded in the period 8–20 June 2017. In the surrounding sectors, smaller rates of displacement and no accelerating trends were observed. The slope was completely stable at lower elevation.

### Failure of a tailings dam embankment at Cadia gold mine (New South Wales, Australia)

At about 6:45 pm on 9 March 2018, a mobile slump affected the southern wall of the Cadia gold mine northern Tailings Storage Facility (TSF). A slurry of sediments, water, and a low level of benign processing reagents were consequently released in the basin of the southern TSF. This adjacent tailings pond maintained its structural integrity, hence preventing the dispersion of waste in the environment. Although there was no major impact on the safety of the workers and on the overall containment capacity of the TSF, operations at the mine had to be halted for several days. The event is just the last of an alarmingly growing list of tailings dam embankment failures that have occurred in recent past, and that occasionally have caused disastrous environmental damages^19^. Whilst it has long been recognized that there is a vast underestimation of the potential of cohesionless soils to undergo rapid failure by static liquefaction, this kind of incidents still takes by surprise engineering designers and practitioners^20^. With regards to the Cadia gold mine northern TSF, it has been suggested that a zone of highly compressible, strain-weakening volcaniclastic material near the foundation level of the southern wall was the controlling factor of the failure. Accelerated lateral movements of the embankment over this weaker layer produced liquefaction (i.e. loss of strength) of the loose saturated tailings, which in turn determined a sudden increase of load on the dam. Full details and analyses are available in the report published by the independent technical review board which investigated the incident^21^. As *in situ* evidences of precarious stability conditions (e.g. tension cracks or minor slumps) may appear with very little notice, the detection of any precursory deformation of tailings dam embankments is essential.

**Figure 3:**
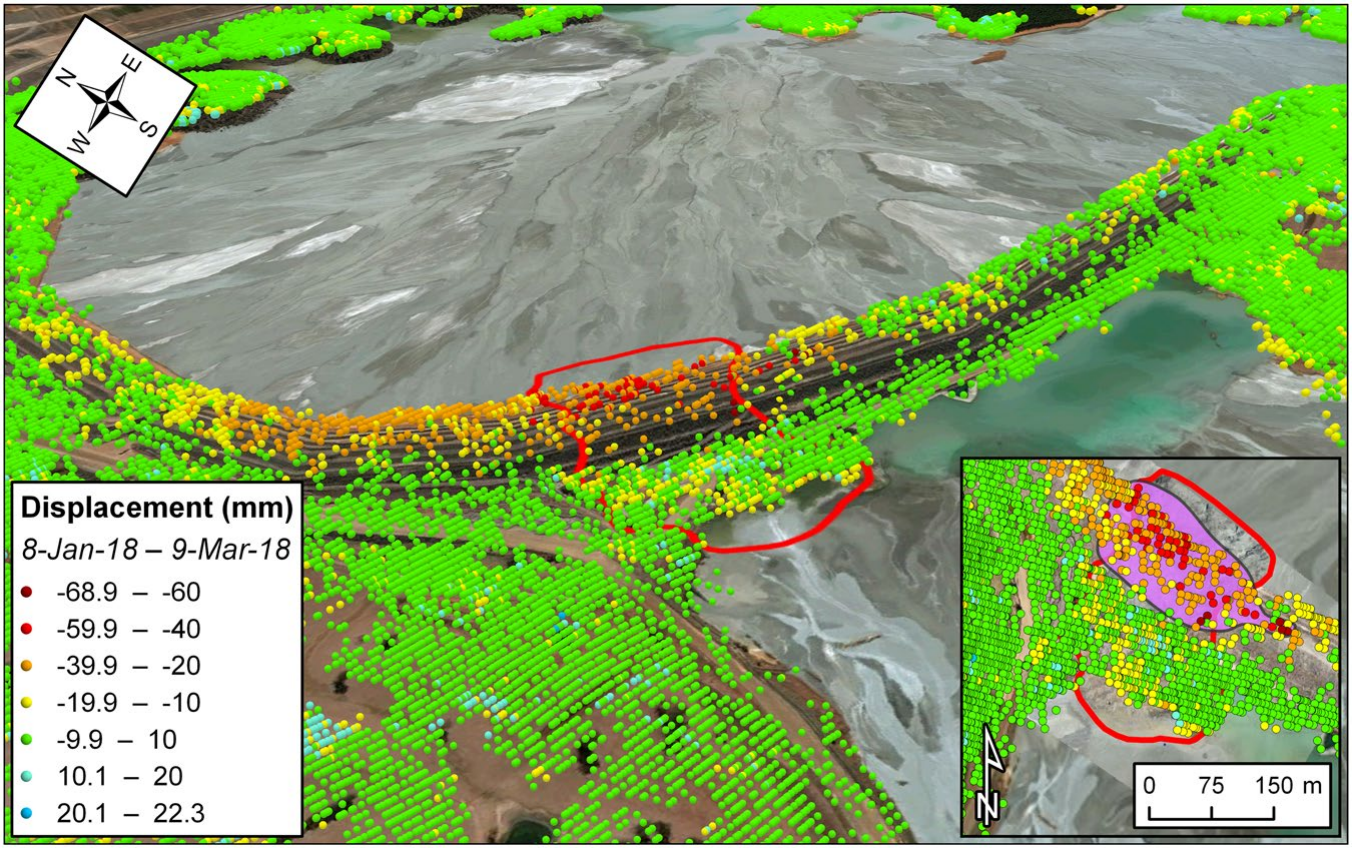
Satellite InSAR data showing precursory deformation leading up to the failure of the Cadia gold mine northern TSF on 9 March 2018. The purple-colored polygon in the inset delimits the area affected by accelerating trends of displacement. The underlying photos in the main figure and in the inset depict pre- and post-failure instants, respectively. The maps with satellite imagery were created with the ArcGIS PRO 2.4 software (https://www.esri.com/en-us/arcgis/products/arcgis-pro/overview).

34 SAR images have been acquired in descending orbit over the mine site between 1 January 2017 and 10 March 2018, with the last acquisition before the event performed on 26 February 2018 (revisit time of 12 days). LOS displacements throughout the southern wall of the northern TSF were very subtle or within the margin of error of the technique for the most part of the monitoring interval, and then substantially increased from January 2018. In particular, the radar targets showing the largest LOS displacements between January–March 2018 (more than 40 mm, and up to 68.9 mm) lie within or in close proximity of the boundaries of the failure (Fig. 3); a maximum LOS displacement of 29.9 mm was measured in the period 14–26 February 2018. Radar targets over the rest of the storage facility showed lower movements or remained essentially stable during the entire monitoring interval. The presence of significant deformation just outside of the eventual failure area may be explained by considering that the weaker volcaniclastic layer diffusely underlay this sector of the northern TSF. On the other hand, it was reported that the embankment was taller and steeper where the slump ultimately developed; this was also where an excavation at the toe was present^21^. It is therefore likely that the structure yielded where the imbalance between the resisting forces and the loading imposed by the liquefaction of the tailings was locally more pronounced.

### Failure predictability

**Figure 4:**
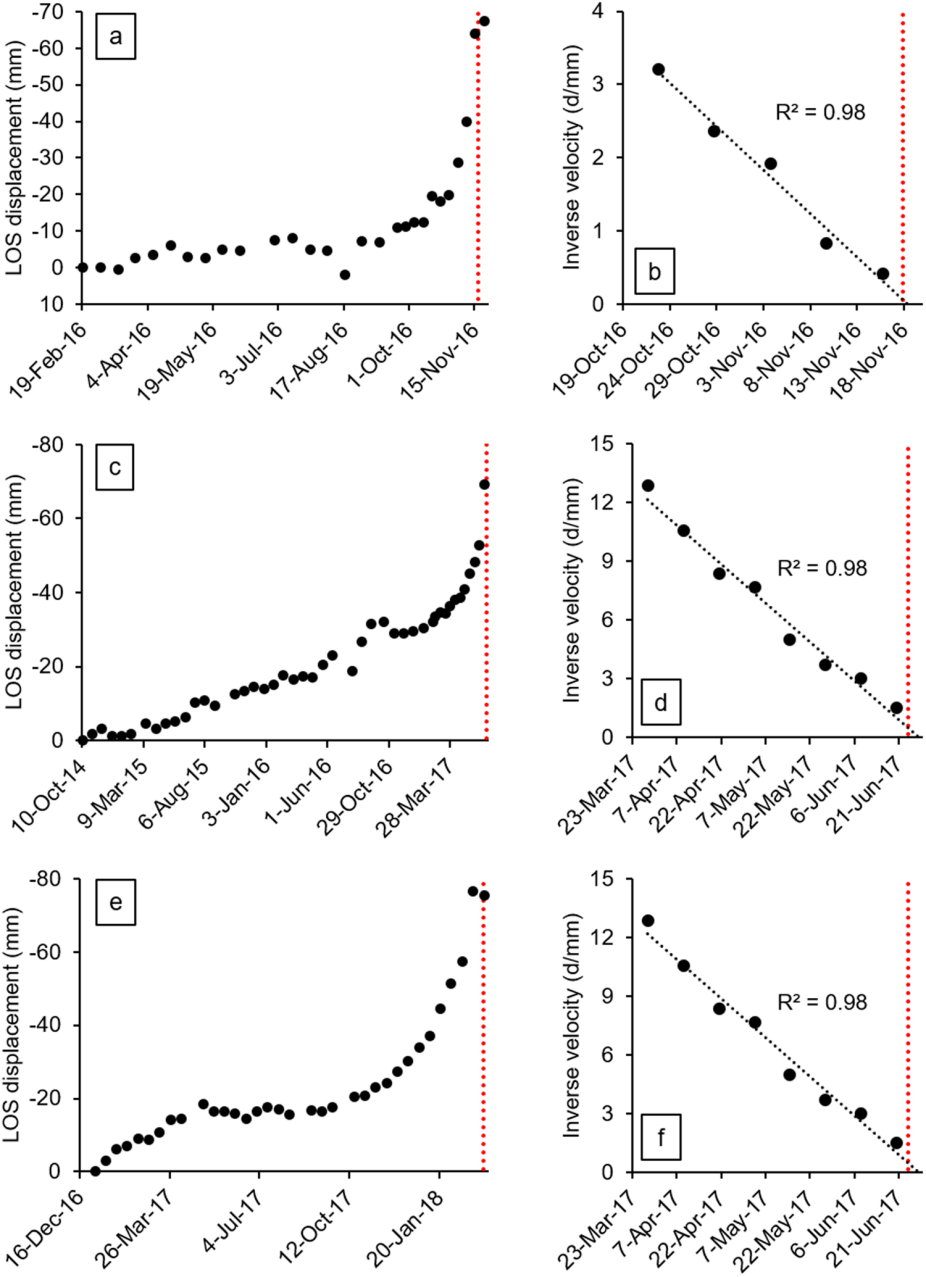
Example of accelerating trend and resulting inverse velocity regression related to (**a**,**b**) the failure of the investigated open-pit mine slope; (**c**,**d**) the Xinmo landslide; (**e**,**f**) the failure of the Cadia gold mine northern TSF. The red dotted lines indicate the actual failure-time.

**Figure 5:**
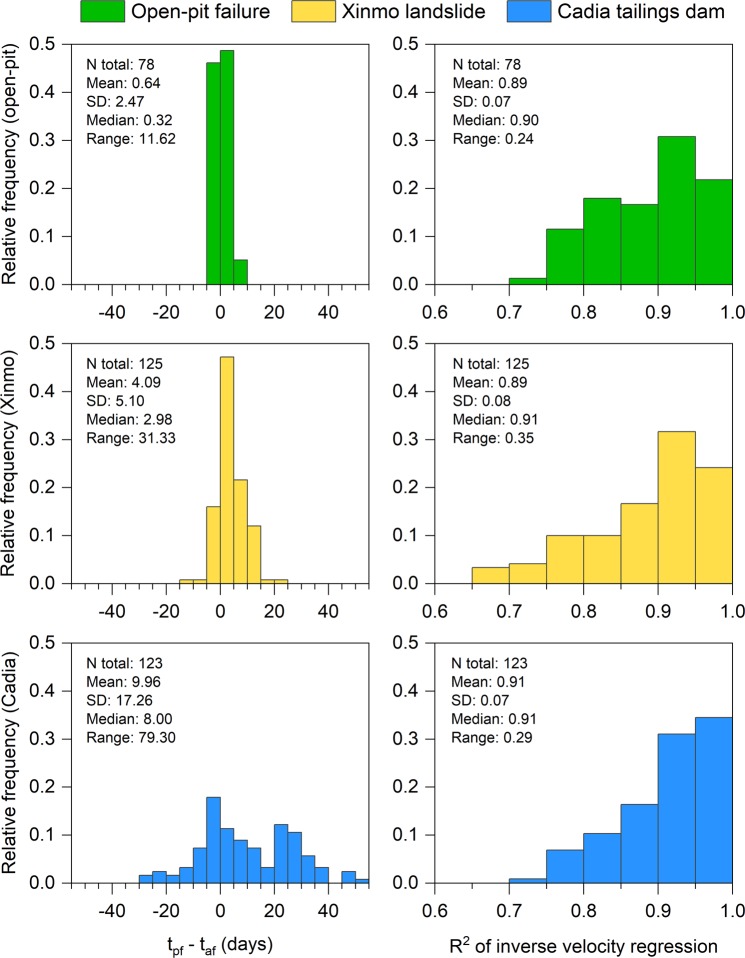
Relative frequency distribution of the errors and of the R^2^ coefficient from the inverse velocity predictions.

The presented datasets reveal accelerating trends of displacement in the weeks leading up to the investigated slope failures; the areas tied with this behavior are enclosed by the purple polygons in the insets of Figs 1–3. Here we back-analyze the detected precursors in order to assess the potential to make effective failure-time predictions if systematic, continuously updated satellite InSAR monitoring campaigns had been carried out. Expected failure-times were derived by applying the inverse velocity method for every radar target showing relevant precursors, and the coefficient of determination R^2^ was taken as a quality index of the regressions. The inverse velocity plots in Fig. 4 show an example of prediction for each case study. The relative frequency distribution of the errors (i.e. *t*_*pf*_ − *t*_*af*_, where *t*_*pf*_ is the predicted time of failure and *t*_*af*_ the actual time of failure) and of the R^2^ values was also computed to provide a measure of the predictive ability that may be deduced from the three stacks of Sentinel-1 images (Fig. 5).

Most of the radar targets within the boundaries of the open-pit slope failure are characterized by a final accelerating trend in the form of a classic tertiary creep curve. Such a phase is already well evident in the early November 2016 acquisitions, roughly two weeks before the event (Fig. 4a,b). A total of 78 radar targets was exploited for the analysis. Linear regression of the inverse velocity data gives a distribution of the predictions that is remarkably centered around the actual time of failure (Fig. 5). The mean error is only slightly more than half a day (where a positive error indicates that the failure occurred earlier than the estimate); the median is off by 0.32 days, and the difference between the earliest and latest expected failure-time is less than 12 days. The R^2^ distribution is left-skewed, with a mean value of 0.9 and a very low standard deviation. All these elements testify that the information contained in the monitoring dataset would have been valuable for predicting the event with high accuracy and more than sufficient forewarning. The time series of 125 radar targets in the source area of the Xinmo landslide also respond to a marked tertiary creep behavior; the onset of the final acceleration phase is typically evident more than one month before the event (Fig. 4c,d). The inverse velocity regressions provide a normal distribution of the errors that is again centered near the actual failure-time (Fig. 5). The mean error is of 4.09 days, and in general the variability is somewhat higher than in the first case study; the total range of the predictions extends for an interval of approximately a month. The R^2^ distribution is instead very similar to what observed in the open-pit dataset. The style of the precursory deformation of the Xinmo landslide is in accordance with the notion that, as high stresses relative to rock strength are produced, large-scale slides are able to accommodate large strain levels for considerable time lengths prior to failure^9^. As a consequence, the event could have been predicted with acceptable accuracy and forewarning notwithstanding the lower frequency of acquisition (12 days) available at the time in the area. Finally, also 123 radar targets within the breach area of the Cadia gold mine northern TSF experienced a monotonic increase of the displacements prior to the event (Fig. 4e,f); these points are all localized on top or on the slope face of the dam (Fig. 3). The reliability of the predictions appears in this instance to be quite lower: the distribution of the errors is visibly flatter, and the tallest bin, while still adjacent to the center of the histogram, stands for a relative frequency of less than 0.2. As a reference, peaks of relative frequency in the open-pit and Xinmo datasets were 0.49 and 0.47, respectively. The gap between the mean prediction and the actual failure-time is about 10 days. The R^2^ distribution is still left-skewed, with mean and standard deviation that are basically equivalent to the other cases. Based on these data, it would have not been possible to pinpoint the time of failure with reasonable confidence.

## Discussion and Conclusions

Several observations may be derived from the results. First of all, the whereabouts of every slope failure are well reflected by the spatial location of the more rapidly moving radar targets. Secondly, the reliability of the time predictions (but not the quality of the inverse velocity regressions) seems to degrade as the gap between the last acquisition before the event and the event itself widens. This is not surprising, as the form of accelerating trends may be influenced by a number of possible changes in background conditions as failure approaches^9^. A lower frequency of acquisition will thus diminish the chances of capturing late trend variations and finding a fit to the inverse velocity curve that is representative of the last step of the failure process. In this sense, the 6-day frequency of acquisition currently offered by the Sentinel-1 constellation may be suitable for singling out firm predictions over relatively short time lengths. Opportune and statistically consistent time predictions could have been extrapolated from the open-pit and Xinmo datasets, with a margin of error of hours in the first case and of a few days in the latter case; this is a very successful outcome when considering the scale of these disasters and the level of risk posed to human life. The same cannot be said about the Cadia gold mine dataset, mainly because the last SAR image was acquired 11 days prior to the incident at the northern TSF. Moreover, the embankment suffered an abrupt rise in external load upon liquefaction of the tailings^21^; as such, a 12-day frequency of acquisition would have been not suitable for tracking the evolution of the escalating failure process. Nevertheless, it may be argued that systematic satellite InSAR monitoring would have still provided decision makers with a definite “qualitative” indication about an ongoing stability issue of the embankment, that could have ultimately produced a breakthrough of the stored material. The magnitude of the precursory deformation detected throughout the southern wall from January 2018 is in fact not in line with the usually very low security tolerances for this type of retaining structures.

To summarize, the experiences herein reported are pioneering yet remarkable examples of how satellite InSAR could improve risk awareness and provide early warning of impending catastrophic slope failures in vast, inaccessible, or otherwise unmonitored regions, with a cost per single slope considerably lower than that required for dedicated monitoring systems. This consideration is only valid for large-scale instabilities undergoing time-dependent development of a controlling release surface, therefore implying a ductile behavior of the slope and extended periods of progressive deformation at relatively slow rates^9^. Typical examples include complex, deep-seated slides responding to rotational, translational, or compound mechanisms. Instabilities of brittle nature, such as rockfalls or small-scale slides in tension or shear in hard rock masses, may not be recorded because of the extreme rapidity with which they transition from a condition of equilibrium to failure^10^. The same limitation applies to shallow landslide types that are activated in the aftermath of a sudden external trigger (e.g. debris flows). In other terms, monitoring and prediction may not be performed when precursors and controlling factors are too instantaneous with respect to the revisit time of the satellite, or over areas that are too small for the spatial resolution of the sensor (5 × 20 m for Sentinel-1). Excessively rapid movements (i.e. more than a few centimeters between consecutive acquisitions) may also generate phase ambiguity or loss of coherence of the interferometric products^11,12^. This issue concerns primarily the terminal stages of tertiary creep, when rates of displacement are more likely to fall out of the range of the technique^22^; the reduction in temporal coherence may even lead to the loss of radar targets^12^. Additionally, as satellite InSAR is capable of tracking only the component of the movement vector projected along the sensor-target direction, a favorable orientation of the slope is required: the true entity of the displacements can be obtained when the slope moves exactly parallel to the LOS, whereas there is no sensitivity with respect to slopes that move perpendicular to the LOS. Finally, information may not be retrieved by means of multi-interferometric analysis over densely vegetated or snow-covered slopes.

## Methods

Radar datasets employed in this study have been acquired by the Sentinel-1 constellation, which is composed of two satellites equipped with C-band (5.6 cm wavelength) SAR sensors featuring a right-looking acquisition geometry and a revisit time of up to 6 days. We employed SqueeSAR, a second-generation InSAR algorithm^13^, in order to process the interferometric images.

SqueeSAR represents the evolution of PSInSAR^23,24^, which is the first technique specifically implemented for the processing of several (at least 15 or more) co-registered, multi-temporal spaceborne SAR images of the same target area. This multi-interferometric analysis is able to provide highly precise ground deformation maps on sparse grids of stable radar targets, called Persistent Scatterers (PS). Once the ratio between the average amplitude of the backscattered radar signal from the observed scene and its standard deviation is established as the so-called “amplitude stability index”, PS are identified in correspondence of values above a predefined threshold of this index. The main characteristics of a PS include high electromagnetic reflectivity, high coherence values, and stable scattering behavior; all these features strongly reduce the occurrence of radar signal decorrelation phenomena. Resolution elements containing a single dominant scatterer with the listed features correspond to a PS. The phase stability associated with these targets during the observation period makes it possible to discern the phase component related to the displacement from the other contributions. While stereoscopic and noise effects can be easily removed, spurious atmospheric effects are strongly correlated in space (within the same SAR scene) but highly decorrelated in time (i.e., among different acquisitions). The atmospheric term is estimated and removed through a statistical analysis of the signals and by applying specific algorithms. Due to their intrinsic features, PS targets generally correspond to buildings, roads, or other man-made structures, hence they are widely available over cities, but are less common in non-urban areas.

The SqueeSAR algorithm partially overcomes this limitation. Not only the Persistent Scatterers are included in the processing analysis, but also the so-called Distributed Scatterers (DS), which correspond to homogeneous areas spread over groups of pixels in a SAR image (rangeland, pasture, bare soils). The application of this new algorithm determines a significant increase in the density of radar targets, ultimately improving the ability to map, monitor and analyze ground deformation in non-urban areas^25–28^. DS are defined through different steps, namely: (i) selection and analysis of image pixels; (ii) statistical comparison of each pixel with the adjacent pixels; (iii) further processing and analysis of statistically homogenous pixels; (iv) identification of DS within statistically homogeneous areas. In particular, the Kolmogorov-Smirnov test is used to detect homogeneous pixels based on the amplitude of the co-registered and calibrated stack of SAR images. Once identified, DS are processed using the PSInSAR algorithm, hence producing the displacement time series of each radar target. The measured displacements of the radar targets are then referred to a stable reference point. Both PSInSAR and SqueeSAR can achieve an accuracy of about 5–6 mm for single measurement, with a geocoding error of few meters^29,30^; these values may vary slightly from point to point, depending on the distance from the reference point and the characteristics of the scatterer. Average velocities are computed through a simple linear regression of the displacement data over the entire monitoring interval (Figs 1–3). Multi-interferometric analyses have a limited capability of measuring rapid movements due to the inherently ambiguous nature of the interferometric phase. The ambiguity related to the discrete sampling interval of the wrapped phase can in fact remain unresolved. The theoretical maximum detectable LOS velocity is usually in the range of some tens of cm/y; it primarily depends on the wavelength and revisit time of the SAR sensor, and secondarily on the specific phase unwrapping technique being used, the spatial pattern of the monitored deformation phenomenon, the density of radar targets, and data noise^12^. An in-depth description of advantages and limitations of the SqueeSAR algorithm, and of multi-interferometric techniques in general, is beyond the scope of this paper. These can be found in other works that are specifically focused on these topics^11,13,14,24^. SqueeSAR has been used to investigate the spatial and temporal distribution of ground deformation in a wide range of fields related to geotechnical engineering, such as landslides^30,31^, slope stability in open-pit mines^32^, subsidence induced by groundwater overexploitation^33^ or mining^34^, assessment of damage from tunneling or other excavation activities^35^, and stability of buildings and infrastructures^36^.

We performed failure-time predictions on radar targets showing relevant precursors by means of the well-known inverse velocity method^7,9,22,37^, which is based on extrapolating the intercept point on the time axis in a plot of inverse velocity versus time (i.e. instant of theoretical infinite velocity). Time series of LOS velocity were smoothed over a 3-point moving average prior to calculation of the reciprocal values. In each regression, in order to use an objective criterion of analysis, we looked for the amount of consecutive data points preceding the failure-time that would produce the highest R^2^ value (typically four to seven data points). In a few instances, the last data point before the actual failure-time was affected by a movement in the opposite direction with respect to the previous trend because of obvious phase wrapping, and was therefore discarded from the regression.

## Data Availability

The raw interferometric products used in this study are freely distributed to the public by the European Space Agency in the framework of the Copernicus Sentinel-1 mission (https://scihub.copernicus.eu/). Data have been processed by means of the SqueeSAR algorithm (patented by TRE ALTAMIRA) and may be available upon reasonable request.

## References

[1] Kilburn CRJ, Petley DN (2003). Forecasting giant, catastrophic slope collapse: lessons from Vajont, Northern Italy. Geomorphology..

[2] Voight B (1988). A method for prediction of volcanic eruptions. Nature..

[3] Voight B (1989). A relation to describe rate-dependent material failure. Science..

[4] Cornelius RR, Scott PA (1993). A materials failure relation of accelerating creep as empirical description of damage accumulation. Rock Mechanics and Rock Engineering..

[5] Kemeny J, Post R (2003). Estimating three-dimensional rock discontinuity orientation from digital images of fracture traces. Computers & Geosciences..

[6] Petley DN, Mantovani F, Bulmer MH, Zannoni A (2005). The use of surface monitoring data for the interpretation of landslide movement patterns. Geomorphology..

[7] Fukuzono, T. A new method for predicting the failure time of a slope. *In Proceedings of the 4th International Conference and Field Workshop on Landslides*, 145–150, (Tokyo 1985).

[8] Petley DN, Bulmer MH, Murphy W (2002). Patterns of movement in rotational and translational landslides. Geology..

[9] Rose ND, Hungr O (2007). Forecasting potential rock slope failure in open pit mines using the inverse velocity method. International Journal of Rock Mechanics and Mining Sciences..

[10] Carlà T (2007). On the monitoring and early-warning of brittle slope failures in hard rock masses: Examples from an open-pit mine. Engineering Geology..

[11] Crosetto M (2016). Persistent Scatterer Interferometry: a review. ISPRS Journal of Photogrammetry and Remote Sensing..

[12] Wasowski J, Bovenga F (2014). Investigating landslides and unstable slopes with satellite Multi Temporal Interferometry: Current issues and future perspectives. Engineering Geology..

[13] Ferretti A (2011). A new algorithm for processing interferometric data-stacks: SqueeSAR. IEEE Transactions on Geoscience and Remote Sensing..

[14] Raspini F (2018). Continuous, semi-automatic monitoring of ground deformation using Sentinel-1 satellites. Scientific reports..

[15] Carlà T (2018). Integration of ground-based radar and satellite InSAR data for the analysis of an unexpected slope failure in an open-pit mine. Engineering Geology..

[16] Fan JR (2017). Geometrical feature analysis and disaster assessment of the Ximno landslide based on remote sensing data. Journal of Mountain Science..

[17] Hu K (2018). New understandings of the June 24^th^ 2017 Xinmo landslide, Moaxian, Sichuan, China. Landslides..

[18] Intrieri E (2018). The Maoxian landslide as seen from space: detecting precursors of failure with Sentinel-1 data. Landslides..

[19] Agurto-Detzel H (2016). The tailings dam failure of 5 November 2015 in SE Brazil and its preceding seismic sequence. Geophysical Research Letters..

[20] Davies, M., McRoberts, E. & Martin, T. Static liquefaction of tailings –fundamentals and case histories. *In Proceedings of Tailings Dams, ASDSO/USCOLD (Las Vegas 2002)*.

[21] Jeffries, M. *et al*. Newcrest ITRB report on NTSF embankment failure. Available at, http://www.newcrest.com.au/media/market_releases/2019/Report_on_NTSF_Embankment_Slump.pdf.

[22] Intrieri E, Carlà. T, Gigli G (2019). Forecasting the time of failure of landslides at slope-scale: A literature review. Earth-Science Reviews..

[23] Ferretti A, Prati C, Rocca F (2000). Non-linear subsidence rate estimation using permanent scatterers in differential SAR interferometry. IEEE Trans. Geosci. Remote Sens..

[24] Ferretti A, Prati C, Rocca F (2001). Permanent scatterers in SAR interferometry. IEEE Transactions on Geoscience and Remote Sensing..

[25] Raspini, F., Moretti, S. & Casagli, N. Landslide mapping using SqueeSAR data: Giampilieri (Italy) case study. *Landslide science and practice* (pp. 147–154). Springer, Berlin Heidelberg (2013).

[26] Meisina Claudia, Notti Davide, Zucca Francesco, Ceriani Massimo, Colombo Alessio, Poggi Flavio, Roccati Anna, Zaccone Andrea (2013). The Use of PSInSAR™ and SqueeSAR™ Techniques for Updating Landslide Inventories. Landslide Science and Practice.

[27] Bardi F (2014). Integration between ground based and satellite SAR data in landslide mapping: The San Fratello case study. Geomorphology..

[28] Bellotti Fernando, Bianchi Marco, Colombo Davide, Ferretti Alessandro, Tamburini Andrea (2013). Advanced InSAR Techniques to Support Landslide Monitoring. Lecture Notes in Earth System Sciences.

[29] Colesanti C, Ferretti A, Prati C, Rocca F (2003). Monitoring landslides and tectonic motions with the Permanent Scatterers Technique. Engineering Geology..

[30] Ciampalini A (2014). Analysis of building deformation in landslide area using multisensor PSInSAR™ technique. International Journal of Applied Earth Observation and Geoinformation..

[31] Cigna, F., Bianchini, S. & Casagli, N. How to assess landslide activity and intensity with Persistent Scatterer Interferometry (PSI): the PSI-based matrix approach. *Landslides*. **10**(3), 267–283.

[32] Paradella WR (2015). Mapping surface deformation in open pit iron mines of Carajás Province (Amazon Region) using an integrated SAR analysis. Engineering Geology..

[33] Cigna F (2012). Monitoring land subsidence and its induced geological hazard with Synthetic Aperture Radar Interferometry: a case study in Morelia, Mexico. Remote Sensing of Environment..

[34] Przyłucka M (2015). Combination of conventional and advanced DInSAR to monitor very fast mining subsidence with TerraSAR-XData: Bytom City (Poland). Remote Sensing..

[35] Bandini A, Berry P, Boldini D (2015). Tunnelling-induced landslides: The Val di Sambro tunnel case study. Engineering Geology..

[36] Bianchini S, Pratesi F, Nolesini T, Casagli N (2015). Building deformation assessment by means of Persistent Scatterer Interferometry analysis on a landslide-affected area: the Volterra (Italy) case study. Remote Sensing..

[37] Carlà T (2017). Guidelines on the use of inverse velocity method as a tool for setting alarm thresholds and forecasting landslides and structure collapses. Landslides.

