# Author Correction: Perspectives on the prediction of catastrophic slope failures from satellite InSAR

**DOI:** 10.1038/s41598-019-55024-x

**Published:** 2019-12-05

**Authors:** Tommaso Carlà, Emanuele Intrieri, Federico Raspini, Federica Bardi, Paolo Farina, Alessandro Ferretti, Davide Colombo, Fabrizio Novali, Nicola Casagli

**Affiliations:** 1 Department of Earth Sciences, University of Florence, Via Giorgio La Pira 4, 50121 Florence, Italy; 2 Geoapp s.r.l., Via Francesco Veracini 30/G, 50144 Florence, Italy; 3 TRE ALTAMIRA, Ripa di Porta Ticinese 79, 20143 Milan, Italy

Correction to: *Scientific Reports* 10.1038/s41598-019-50792-y, published online 01 October 2019

In Figure 4, panel F (Cadia case study) is erroneously a duplicate of panel D (Xinmo case study). The correct Figure 4 appears below as Figure 1.

**Figure 1:**
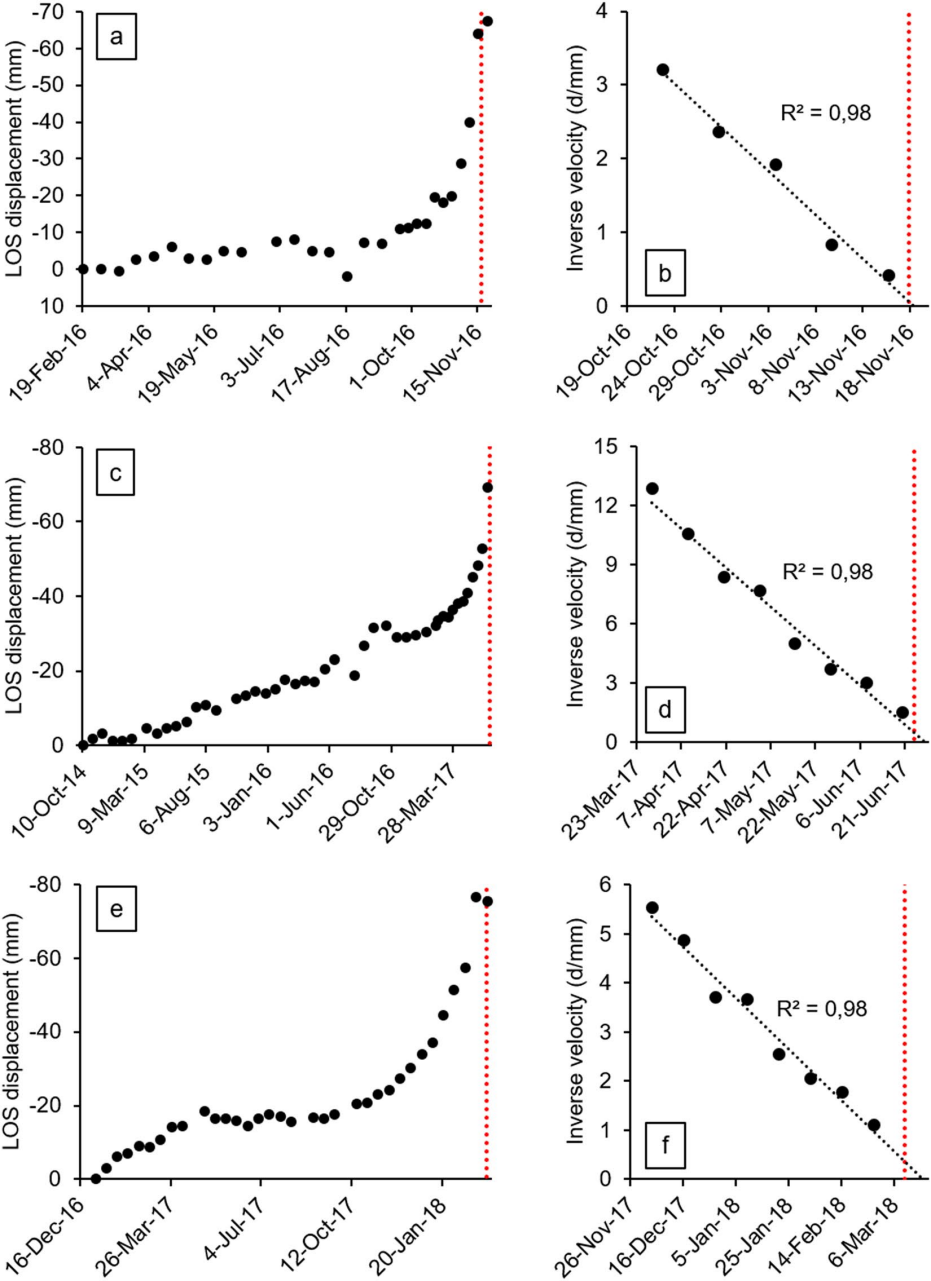
.

